# The Interferon-Gamma Release Assay versus the Tuberculin Skin Test in the Diagnosis of *Mycobacterium tuberculosis* Infection in BCG-Vaccinated Children and Adolescents Exposed or Not Exposed to Contagious TB

**DOI:** 10.3390/vaccines11020387

**Published:** 2023-02-07

**Authors:** Magdalena Druszczynska, Michal Seweryn, Sebastian Wawrocki, Anna Pankowska, Magdalena Godkowicz, Magdalena Kowalewska-Pietrzak

**Affiliations:** 1 Department of Immunology and Infectious Biology, Institute of Microbiology, Biotechnology and Immunology, Faculty of Biology and Environmental Protection, University of Lodz, Banacha 12/16, 90-237 Lodz, Poland; 2 Biobank Laboratory, Department of Molecular Biophysics, Faculty of Biology and Environmental Protection, University of Lodz, Banacha 12/16, 90-237 Lodz, Poland; 3 Regional Specialized Hospital of Tuberculosis, Lung Diseases, and Rehabilitation in Lodz, Okolna 181, 91-520 Lodz, Poland

**Keywords:** tuberculosis (TB), latent *Mycobacterium tuberculosis* infection (LTBI), Bacillus Calmette–Guérin (BCG) vaccination, tuberculin skin test (TST), interferon-gamma release assay (IGRA)

## Abstract

Background: Children have an increased risk of developing active tuberculosis (TB) after exposure to *Mycobacterium tuberculosis* (*M.tb*), and they are more likely to develop the most severe forms of TB. Rapid diagnosis and treatment of latent *M.tb* infection (LTBI) is essential to lessen the devastating consequences of TB in children. Objective: The aim of the study was to evaluate TST (tuberculin skin test) and IGRA (interferon-gamma release assay) utility in identifying LTBI in a cohort of Bacille Calmette–Guérin (BCG)-vaccinated Polish children and adolescents exposed or not exposed to contagious TB. In addition, we asked whether quantitative assessment of IGRA results could be valuable in predicting active TB disease. Results: Of the 235 recruited volunteers, 89 (38%) were TST-positive (TST+), 74 (32%) were IGRA-positive (IGRA+), and 62 (26%) were both TST+ and IGRA+. The frequency of TST positivity was significantly higher in the group with (59%) than without TB contact (18%). The percentage of TST+ subjects increased with age from 36% in the youngest children (<2 years) to 47% in the oldest group (>10 years). All positive IGRA results were found solely in the group of children with TB contact. There was a significant increase in the rate of positive IGRA results with age, from 9% in the youngest to 48% in the oldest group. The 10 mm TST cutoff showed good sensitivity and specificity in both TB exposed and nonexposed children and was associated with excellent negative predictive value, especially among nonexposed volunteers. Mean IFN-γ concentrations in IGRA cultures were significantly higher in the group of LTBI compared to the children with active TB disease, both TST+ and TST−. Conclusions: Both TST and IGRA can be used as screening tests for BCG-vaccinated children and adolescents exposed to contagious TB.

## 1. Introduction

The World Health Organization (WHO) estimates that one million children suffer from tuberculosis (TB), and about 250,000 die from the disease each year. Children make up one-tenth of the people worldwide who develop TB, but confirming diagnosis in children is difficult, resulting in serious delays in initiating treatment. Despite different diagnostic possibilities, many cases of pediatric TB remain undetected. TB diagnosis in children is challenging and unreliable due to problems with specimen collection and microbiological confirmation of *Mycobacterium tuberculosis* (*M.tb*) infection. Due to the variety of its clinical presentations and the lack of particular signs and symptoms, TB is difficult to diagnose in the first few years of life [1,2].

In recent decades, TB control strategies have focused almost exclusively on identifying and treating active *M.tb* infections. However, it is evident that this approach alone is not sufficient to combat TB, given that the majority of new TB cases are the consequence of reactivation of latent *M.tb* infection (LTBI). LTBI is defined as a state of persistent immune response to stimulation by *M.tb* antigens with no evidence of clinically manifest active TB [3]. Recent estimates indicate that approximately one-quarter of the world’s population is latently infected with *M.tb* [4,5]. The latency period is variable, and healthy individuals can carry LTBI for up to a lifetime constituting a major reservoir for new cases of active TB. Reactivation, the conversion of subclinical latent infection into active disease, most often affects adults or adolescents, but can also occur in children, explained by the immaturity of their immune system [6]. Understanding the causes of LTBI reactivation is incomplete, but includes bacterial, host, and environmental factors. Early diagnosis of LTBI is crucial to ensure prompt treatment and reduce the risk of developing active TB. Children exposed to TB have a high risk of *M.tb* infection, which increases with the duration of the contact. The risk of developing active TB following infection for children is 10–20%, of whom 5% are likely to develop the disease within the first 12 months after primary *M.tb* infection and another 5% in the remainder of their lifetime [7]. Epidemiological data show that clinical TB can be developed by approximately 40% of *M.tb*-infected infants, 24% of children between 1 and 10 years, and 16% of adolescents in 11–15 years of age, if anti-tuberculous treatment is not implemented [7].

However, there is currently no diagnostic method to assess the risk of developing active TB in latently infected individuals, including children [8]. At present, two tests for LTBI diagnostics are available, the tuberculin skin test (TST) and the interferon-gamma (IFN-γ) release assay (IGRA). Both are based on the detection of a specific immune response against *M.tb* antigens after a contact with the pathogen. TST is one of the oldest diagnostic methods, having been used in clinical medicine since 1910. The test involves injecting a standard dosage of tuberculin units intradermally into the patient’s forearm, and, after 48–72 h, the diameter of skin induration in millimeters is measured to determine the presence of type IV delayed hypersensitivity (DTH) at the injection site [9,10]. The correct interpretation of TST requires consideration of potential factors that may limit its clinical applicability, such as interindividual variability of the tuberculin response, infection with nontuberculous mycobacteria (NTM), or, most commonly, prior immunization with the bacillus Calmette–Guérin (BCG) vaccine [11]. In 2021, 100 years passed since the BCG vaccine was finally developed at the Pasteur Institute in Lille, France and began to be used in the prevention of TB. In Poland, the first vaccination with the Brazilian BCG strain Moreau was carried out in 1926, and, in 1956, it was introduced as a compulsory vaccination in the National Immunization Program [12]. A number of BCG revaccinations were once part of the Polish vaccination schedule for kids and teenagers, with the first dosage administered to infants up to 1 month of age and further doses administered at 2, 4, 7, 12, 15, and 18 years of age. The number of BCG doses was reduced in the 1990s, and, since 2006, only newborns who have attained a body weight of 2000 g and do not have any contraindications to vaccination receive a single dose of BCG within the first few days of life, in accordance with WHO recommendations [13]. As long as the epidemiological indicators of TB in Poland are unsatisfactory, BCG vaccination will remain routine for the entire population of Polish children. Prior BCG vaccination is a major factor confusing the interpretation of TST results, especially in countries such as Poland, where the vaccination rate is high and the TB prevalence is low [14]. Thus, there is a need for alternative diagnostic assays to identify LTBI, which include IGRA tests. They are blood-based tools that detect IFN-γ produced by lymphocytes upon stimulation ex vivo with *M.tb*-specific peptide antigens, ESAT-6 (6 kDa early secretory antigenic target), CFP-10 (culture filtrate protein 10), and Tb7.7 (p4), which are not present in any BCG strain or most environmental mycobacteria. Therefore, interpretation of IGRA results, which is based on measuring the number of IFN-γ releasing T cells or the amount of IFN-γ released is not affected by BCG vaccination or NTM infection. However, IGRAs are known to show limited sensitivity in immunocompromised individuals and young children. Moreover, IGRAs are unable to differentiate between active TB and latent *M.tb* infection [15].

Although the incidence of TB in Poland is steadily declining, it remains high especially in the eastern part of the country. According to WHO data, the incidence of TB in 2020 was 9.6 per 100,000 population, despite high BCG coverage reaching 92% [4,16]. TB incidence rates increased with age group from 1.4/100,000 among children up to 14 years of age to 2.6/100,000 in those between 15 and 19 years of age [17], requiring effective identification and treatment of adolescents with LTBI. As far as we are aware, studies of the effectiveness of TST and IGRA in diagnosing LTBI in Poland have been limited to examining healthcare workers exposed to virulent *M.tb* [18,19,20]. Therefore, our aim was to evaluate TST and IGRA utility in identifying LTBI in a cohort of BCG-vaccinated Polish children and adolescents exposed or not exposed to contagious TB. Although the absence of a gold standard for *M.tb* infection is acknowledged, we believed that a negative IGRA test result would serve as a stand-in for the absence of *M.tb* sensitization. In addition, to address the question of whether quantitative assessment of IGRA results can be valuable in predicting active TB disease, an analysis was also performed according to this objective.

## 2. Materials and Methods

### 2.1. Study Design and Inclusion Criteria

The investigation was carried out within a project funded by the National Science Center, grant number 2016/21/B/NZ7/01771. The protocol for the cross-sectional study was approved by the Research Ethics Committee of the Medical University in Lodz (no. RNN/138/15/KE).

The study was conducted in patients of Polish origin hospitalized between January 2017 and December 2019 at the Regional Specialized Hospital of Tuberculosis, Lung Diseases and Rehabilitation in Lodz. Participants were included in the study who (i) were younger than 18 years of age, (ii) had been vaccinated at birth with *Mycobacterium bovis* BCG Moreau vaccine according to the Polish national immunization program, (iii) were HIV-negative, and (iv) had undergone IGRA (QuantiFERON-TB Gold Plus assay) and TST testing. On the basis of a detailed interview a close contact with acid-fast bacilli (AFB) smear-positive and subsequently culture-confirmed source *M.tb* cases was confirmed/excluded. We used the definition of “close contacts” proposed by Behr et al., which is at least a 40 h exposure to TB in a closed room [21]. Subjects were excluded from further analysis if they (i) had a previous history of active TB, (ii) were receiving treatment with steroids or other immunosuppressive drugs, (iii) had etiological evidence of infection with virus, mycoplasma or bacteria other than *M.tb*, or (iv) had not been tested with IGRA and TST. A total of 235 participants, aged 1–17, were included in the study. Of these, 115 were confirmed close contacts of active TB patients (TB contact group), and 120 participants did not report prior exposure to active TB (no TB contact group). Active TB was further screened in individuals with (i) a history of exposure to active TB, and (ii) clinical and radiographic evidence consistent with TB. For the diagnosis of TB, children’s gastric aspirates or bronchoaspirates were used and examined using standard microbiological techniques such as Ziehl–Neelsen staining (smear), culture on liquid (BACTEC MGIT 960 system) and solid media (Löwenstein–Jensen), and genetic testing using the GeneXpert MTB/RIF molecular system. If children received a diagnosis of LTBI, or active TB, they ended follow-up in our study. All children with negative IGRA result returned 8 weeks after first hospitalization for repeat testing. Children were considered healthy if the repeat IGRA test after 8 weeks was negative and subsequent chest X-rays was normal.

Demographic characteristics of the study cohort are shown in Table 1. There was a significant difference between the TB and no TB contact group in terms of age (Mann–Whitney U test, *p* = 0.01); however, there was no differences between regarding the sex rate (χ^2^ test, *p* = 0.95) (Table 1). The percentage of the youngest participants in the study (<2 years) was significantly higher in the group of volunteers with TB contact than without TB contact (χ^2^ test, *p* = 0.0006) (Table 1).

### 2.2. Interferon-Gamma Release Assay (IGRA)

Samples of peripheral blood were taken from all volunteers prior to the tuberculin skin testing to perform QuantiFERON-TB Gold Plus (Qiagen, Hilden, Germany) assay. IGRA was assessed at baseline in all volunteers and repeated after 8 weeks when the result was negative. The sampling and analyses of the test were performed according to the instructions of the manufacturer. Four distinct collection tubes—a Nil Control tube, a TB1 Antigen tube, a TB2 Antigen tube, and a Mitogen (positive control) tube—were each filled with 1 mL of blood. The collecting tubes were then centrifuged at 2500 RCF for 15 min after being incubated at 37 °C for 16–20 h. By using ELISA and comparing the results to a concentration standard, the interferon-gamma (IFN-γ) level in the resultant plasma in the tubes was determined. Using a multifunctional counter Victor 2 (Wallac Oy, Turku, Finland) equipped with a 450 nm filter, the optical density (OD) of each sample was determined. To interpret the results, a standard curve of human recombinant IFN-γ was prepared on the basis of the mean absorbance values of two replicates of each standard dilution. The value of the IFN-γ concentration in each test sample was determined on the basis of the standard curve prepared for each assay using the QFT-Plus Analysis Software version 2.71.2. According to the producer, QFT-Plus is positive when TB1 Antigen-Nil and TB2 Antigen-Nil are ≥0.35 IU/mL. A participant was classified as IGRA-positive (and, thus, assumed to be infected with *M.tb* for the purpose of this analysis) if either the baseline or 8 week IGRA was positive. Although it is recognized that there is no gold standard for *M.tb* infection, we considered that a negative IGRA test result would serve as a stand-in for the absence of *M.tb* sensitization. Children with negative IGRAs at both timepoints were considered uninfected.

### 2.3. Tuberculin Skin Test (TST)

First, 0.1 mL containing 2 tuberculin units (TU) of purified protein derivative (PPD) RT 23 (Statens Serum Institut, Copenhagen, Denmark) was injected intracutaneously into the dorsal side of the lower arm. The diameter of the skin induration was measured after 72 h. A result was considered positive when the size of the induration was ≥10 mm. The induration was read by trained nurses. A second reading was performed by another person to confirm the result if it was on the borderline. If there was disagreement, the TST was read a third nurse, and the consensus result was applied. TST was defined as being positive if the diameter of skin induration was ≥10 mm, in line with the guidelines of the Polish Respiratory Society.

### 2.4. Statistical Analysis

Statistical analysis of the results was performed using Statistica 13.3 PL software (Statsoft) The nonparametric chi-square (χ^2^) test or Fisher’s exact test was used to assess the statistical significance of the differences in frequency. Differences in IFN-γ concentrations were assessed using the Mann–Whitney U test. A *p*-value < 0.05 was considered significant. Interpretation of the IGRA test result was performed using QuantiFERON-TB Gold Plus Software. The binomial distribution was used to determine confidence intervals (95% CI). MedCalc^®^ diagnostic calculator was used to determine sensitivity, specificity, positive prediction value, and negative prediction value for the TST in correctly identifying IGRA-positive children (https://www.medcalc.org/calc/diagnostic_test.php, accessed on 29 November 2022). Utilizing Cohen’s kappa (κ) coefficients, the degree of agreement between the TST and IGRA test results was determined (www.graphpad.com/quickcalcs/kappa1/, accessed on 29 November 2022). κ values above 0.8 showed great agreement, values between 0.8 and 0.4 represented good correlation, and values below 0.4 indicated poor agreement.

## 3. Results

### 3.1. TST and IGRA Results

**Figure 1:**
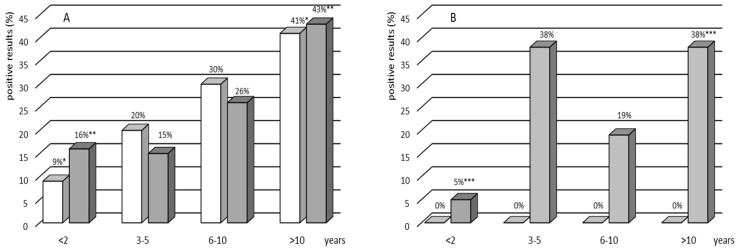
Percentage of the IGRA-positive (white bars) and TST-positive (gray bars) results by age group in the group with (**A**) and without (**B**) TB contact. Asterisks indicate a statistically significant difference at *p* < 0.05. A nonparametric chi-square test or Fisher’s exact test was used to assess the statistical significance of differences in the prevalence of positive TST and IGRA results within an age group.

Of the 235 volunteers, 113 (48%) had a TST size <5 mm, 33 (14%) had a TST size from 5 to 9 mm, and 89 (38%) had a TST size ≥10 mm. The median size of the TST was 5 mm. The frequency of TST positives was significantly higher in the TB contact (59%) than no TB contact (18%) group (*p* = 0.00001, χ^2^ test) (Table 2). The percentage of TST-positive results when using the cutoff ≥ 5 mm was 75% (86/115) or 30% (36/120) in the TB contact or no TB contact group, respectively (*p* = 0.00001, χ^2^ test). This percentage did not significantly differ from the percentage designated as positive using a cutoff ≥10 mm. Twenty-two (19%) and five (5%) volunteers from the TB contact and no TB contact groups, respectively, showed a strong positive response to tuberculin with a skin induration diameter of greater than 15 mm. The percentage of TST positive results when using the cutoff of 5 mm was 75% (86/115) or 30% (36/120) in TB contact or no TB contact children, respectively (*p* = 0.00001, χ^2^ test). This percentage did not significantly differ from the percentage designated as positive using a 10 mm cutoff. Twenty-two (19%) and 5 (5%) volunteers from the TB contact and no TB contact groups, respectively, showed a robust positive reaction to PPD with a skin induration diameter of greater than 15 mm (Table 2). The proportion of children with positive TST results increased significantly from 16% in the youngest age group (<2 years) to 43% in the oldest age group (>10 years) (*p* < 0.005, χ^2^ test) in the group of volunteers exposed to TB and from 5% to 38% in the group of children without TB contact (Figure 1). Analysis of the results of skin reactions to tuberculin with a 10 mm cutoff in four age ranges of study volunteers (<2 years, 3–5 years, 6–10 years, and >10 years) showed a positive correlation between the age and development of DTH to the subcutaneously injected PPD (Spearman’s rank correlation *p* = 0.05; r = 0.12).

There were no indeterminate IGRA results in any of the groups. Of the 235 children recruited, 74 (32%) were IGRA-positive (Table 2). All positive IGRA results were found solely in the group of children with TB contact: 13 in the group of children with active TB and 61 in the LTBI group (Table 2). The rate of IGRA positives showed a significant increase with age in both studied cohorts, with and without TB contact (Figure 1). The analysis of IGRA results in the age groups showed a significant increase in the rate of IGRA positives, from 9% among children younger than 2 years old to 48% among those older than 10 years (*p* = 0.02, χ^2^ test) (Figure 1).

### 3.2. Sensitivity, Specificity, Positive Predictive Value (PPV), and Negative Predictive Value (NPV) for TST in Correctly Identifying IGRA Positivity in TB Exposed and Unexposed Subjects

Using the assumption that a positive IGRA is equivalent to LTBI, we assessed the overall sensitivity, specificity, and positive and negative predictive values for the TST in TB contact and no TB contact group. Sensitivity, specificity, PPV, and NPV for skin TST for diagnosing LTBI in TB exposed subjects were 91%, 75%, 84%, and 85%, respectively, while the corresponding values in TB unexposed group were 100%, 83%, 0%, and 100%, respectively (Table 3 and Table 4).

### 3.3. TST and IGRA Agreement

Utilizing Cohen’s kappa (κ) coefficients, the degree of agreement between the TST and IGRA test results was also determined. The overall agreement between the TST and IGRA in all studied volunteers was good (κ = 0.635) (Table 5). In the entire study cohort, 62 (26%) volunteers were IGRA-positive and TST-positive at the same time, all from the group of children in contact with TB, including 13 in the group of children diagnosed with active TB and 49 among volunteers with LTBI (Table 5). Three other patients with microbiologically confirmed TB were TST-negative and IGRA-negative (Supplementary Table S1). In contrast, 83% of the children without TB contact vs. 30% TB contact volunteers had negative results for both TST and IGRA (*p* < 0.00001, χ^2^ test). Similarly to the rate of TST-positive/IGRA-positive results, the percentage of TST-positive/IGRA-positive results was significantly higher in the youngest children compared to the oldest age group (*p* = 0.01, χ^2^ test) (Table 6). The majority of discordant results in the study cohort were due to TST-positive and IGRA-negative results (*n* = 27; 12%), six in the group of children with TB contact and 21 in the group of children without TB contact (Table 5).

### 3.4. IFN-γ Responses to M.tb Specific Antigens

**Figure 2:**
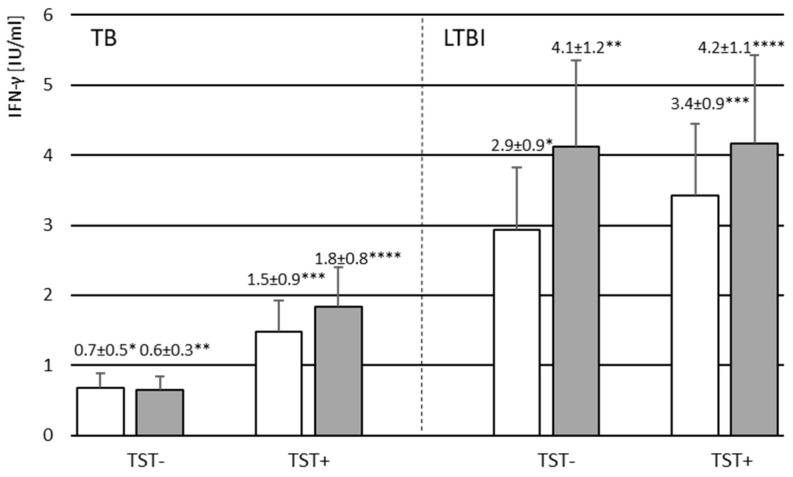
The mean level of IFN-γ produced by leukocytes following stimulation with *M.tb* antigens in TB1 (white bars) and TB2 (gray bars) QuantiFERON TB Plus whole blood cultures in the groups of TB and LTBI volunteers with negative (TST−) or positive (TST+) reaction to tuberculin. Abbreviations: LTBI—latent *M.tb* infection, TB—tuberculosis, TST—tuberculin skin test. * *p* = 0.014; ** *p* = 0.014, *** *p* = 0.011, **** *p* = 0.018. The nonparametric Mann–Whitney U test was used to assess the statistical significance of differences in IFN-γ concentrations in TB1 and TB2 blood cultures between TB and LTBI groups.

The amounts of IFN-γ produced in response to *M.tb* specific antigens in blood cultures from the studied volunteers were also analyzed. Results are presented as the difference in the level of cytokine produced by leukocytes after stimulation with *M.tb* antigens in TB1 and TB2 QuantiFERON TB Plus whole-blood cultures and baseline Nil level. Mean concentrations of IFN-γ in TB1 and TB2 blood cultures were significantly higher among the TB contacts compared to those without TB contact, both TST-positive and TST-negative (Supplementary Figure S1). There was no difference in the mean IFN-γ levels measured in both *M.tb* antigens stimulated cultures among the TST-positive and TST-negative volunteers (Supplementary Figure S1). The concentration of IFN-γ produced was not correlated with the age of the studied volunteers (Supplementary Figure S2). As indicated by the results in Table 7 and Figure 2, IFN-γ levels after *M.tb* stimulation were significantly higher in the group of LTBI volunteers compared to the children with active TB disease (TB1 culture *p* = 0.005, TB2 culture *p* = 0.0007, Mann–Whitney U test), both TST-positive and TST-negative. A positive correlation between the IFN-γ levels and the TST induration size was found in both TB1 (Spearman’s rank correlation coefficient r = 0.617; *p* < 0.0001) and TB2 (Spearman’s rank correlation coefficient r = 0.626; *p* < 0.0001) whole-blood cultures (Supplementary Figure S3).

## 4. Discussion

Thousands of children worldwide are exposed to *M.tb* each year, yet it is still unclear how effective contact testing is. To our knowledge, our study is one of the few conducted in Poland on the proportion of LTBI, the first performed among BCG-vaccinated children and adolescents exposed or not exposed to contagious TB, for which purpose we used TST and IGRA. Although there is no gold standard for the diagnosis of LTBI, we compared IGRA with TST to determine the percentage of LTBI in our study cohort.

In the entire study group, the proportion of positive IGRA was 32%, similar to data reported in studies conducted in other countries [22,23,24,25]. All positive IGRA results were found only in the group of children with confirmed TB contact, showing a correlation with the exposure risk. The percentage of TST-positive results found (38%) was slightly higher than for IGRA: 59% among volunteers with TB contact and 18% among volunteers without TB contact. Our findings showed a rate of TST-positive/IGRA-positive results of 26% among all volunteers, while the rate of TST-negative/IGRA-negative results reached 57%. However, the proportion of TST-negative/IGRA-negative results was significantly higher among volunteers not exposed to TB (82%) than children with confirmed TB contact (30%). These findings are in line with the results of previous research enrolling TB contacts [26,27,28]. Children with both positive IGRA and TST results tend to have clinically confirmed LTBI. Consistently, in our study, children with IGRA-positive/TST-positive results were associated with a TB contact risk, suggesting that IGRA-positive/TST-positive children should be assessed for TB prophylactic treatment.

Consistent with our results, TB contact children showed a higher percentage of positive IGRA results, but a lower percentage of positive TST results, compared to those without contact with TB patients. IGRAs have been shown to provide higher specificity than TST [29]. Some investigators have indicated that the TST is more sensitive than IGRA, although less specific, in the BCG vaccinated population [29,30]. A systematic meta-analysis by Diel et al. showed that the specificity of IGRA ranged from 98% for the T-SPOT^®^.TB to 100% for the QuantiFERON TB Gold IT, while the TST specificity ranged from 55% to 95% [31]. In another meta-analysis based on 20 studies, Pai et al. showed that the sensitivity and specificity of TST were heterogeneous, with a pooled estimate of 77% (sensitivity) and 59% (specificity) in the BCG-vaccinated population, which, according to the authors, indicates a major limitation of the TST due to the possibility of cross-reactions [10].

The overall concordance between TST and IGRA results found in our study was significant, which is in agreement with the results of other research groups [32]. When there was a discrepancy between TST and IGRA results, children with TB contact were more likely to be TST-negative and IGRA-positive, whereas children without TB contact were more likely to be TST-positive and IGRA-negative, which may be primarily related to the effect of BCG vaccination on TST results. This suggestion may be supported by the results of Ferrra et al. and Diel et al., who noted poor concordance between TST and IGRA results among BCG-vaccinated individuals, but much higher in those who had not been vaccinated [33,34]. Data from a Canadian study that evaluated the effect of neonatal BCG vaccination on the TST size in preschool aboriginal children living on a reserve showed that the TST result is affected by the cutoff point and age [35]. Reid et al., using a cutoff point of 5 mm, found that positive TST reactions were more common in children of all ages vaccinated with BCG at birth, but the frequency of TST positives in immunized children older than 1 year did not differ using a cutoff of 15 mm. Using a cutoff of 10 mm, more vaccinated children had a positive result of TST at the age of 1 year, but no differences were observed until 4 years of age [35]. According to a meta-analysis by Farhat et al., if the vaccination was administered at birth, the effect of the vaccine on the TST size was reduced. Less than 1% of children had a TST of 10 mm or more by the age of 10, but if BCG was administered beyond the first year of life, a much higher proportion of children maintained TST-positive [36]. Researchers from Spain came to a similar conclusion when they discovered that, 3 years after receiving the immunization, the chance of TST results being falsely positive due to the administration of BCG at birth disappeared [9].

The analysis of TST and IGRA results in selected age groups revealed that the tests were significantly more often positive in the oldest age group (>10 years) compared to the younger one (<2 years). These results seem to be in agreement with epidemiological TB data in Poland. In the same period, the incidence rates of TB in general population grew along with the age group from 1.2 per 100,000 among children (0–14 years) to 3.6 per 100,000 among subjects in the age group 15–19 years [17].

Another important finding of this study is that, among 115 participants with TB contact, we identified 62 (54%) children with IGRA-positive/TST-positive results and 12 (11%) subjects with IGRA-positive/TST-negative results. It is thought that genetic variation and differences in immune response may partly explain why some people develop a delayed hypersensitivity reaction to PPD, while others do not react at all. Several epidemiological studies have found high levels of heritability for TST reactivity and have linked genetic variants to TST negativity or positivity [37,38]. Interestingly, some people living in highly TB endemic areas display persistent lack of TST reactivity, suggesting that these individuals are likely to be naturally resistant to *M.tb* infection rather than deficient in eliciting the DTH response [38]. Our previous study showed that CD14(−159C/T) polymorphic variants may be one of the genetic determinants of the development of DTH to PPD in Polish individuals subjected to BCG immunization [39]. In addition to the genetic background and immune status, factors such as age, malnutrition, the interval between exposure to the antigen and the test performance, and cross-reactivity with environmental nontuberculous mycobacteria or other pathogens may influence the results of TST [40,41,42,43]. Despite its drawbacks, TST remains the most widely used technique in the diagnosis of LTBI because of its simplicity and in vivo evidence of a cellular immune response against mycobacteria.

The correct interpretation of screening tests should be taken into account by those who treat pediatric TB. There will always be a tradeoff among test sensitivity, specificity, PPV, and NPV because no test is ever completely accurate. In our study cohort, among children exposed to contagious TB, TST had a PPV of 84% and an NPV of 85%, while, among those not exposed, the PPV and NPV values were 0% and 100%, respectively. False-positive and false-negative results, however, both have therapeutic and financial repercussions. Mathematical modeling suggests that not only treatment of LTBI in children may be a highly cost-effective strategy, but also screening for exposure and treatment without testing for evidence of infection [44]. However, since children, particularly the youngest ones, are at the highest risk of progression of infection to disease and are also the most susceptible to severe disseminated forms of TB, for many clinicians, the greater sensitivity of the screening test and high NPV are more important than its specificity and PPV when assessing children in this age group. The WHO recommends treating all children under 5 years of age for LTBI after significant exposure to an infectious case of TB, regardless of the diagnostic test result and BCG vaccination status [45].

As both tests, TST and IGRA, are based on the assessment of the immune response of sensitized lymphocytes and activated antigen-presenting cells to specific *M.tb* antigens, we aimed to correlate the magnitude of TST induration and the level of IFN-γ produced in IGRA blood cultures. In our study, in line with the results of others [18,19], a positive correlation was observed between both results. This confirms that, although both tests do not measure the same components of the immune response, they are a part of the multifaceted host response to *M.tb* is influenced by numerous mediators with pleiotropic inflammatory effects. Many authors suggest that quantitative analysis of the IFN-γ may be a helpful indicator in monitoring the risk of developing active TB in latently infected people [46,47]. As indicated by the results in our study, the concentration of IFN-γ produced in response to specific *M.tb* antigens in the IGRA cultures of children was significantly higher in the LTBI group than in TB patients. The literature data show that assessing the level of IFN-γ produced in response to *M.tb* antigens may be of particular importance, especially in individuals with a high baseline IFN-γ level, which may indicate recent mycobacterial infection. The risk of developing clinical TB in the 1–2 years following exposure was 10 times higher in those who had a robust IFN-γ response to the ESAT-6 antigen than in people who had a weak one [34]. This finding was in line with the observation that effective anti-tuberculous treatment reduced the amount of IFN-γ produced in response to ESAT-6 and CFP-10 [48]. In a close TB contact in the Gambia, who developed active disease over a 5 month period, growing qualitative and quantitative ELISPOT counts of IFN-γ-producing T cells in response to ESAT-6/CFP-10 peptides were found [34]. In addition, studies in mice have shown a correlation between T-cell responses to ESAT-6 and CFP-10 *M.tb* antigens, in vivo mycobacterial replication, and TB progression [49]. It appears that enhanced IFN-γ production in response to ESAT-6 and CFP-10 in LTBI patients may be able to foretell the development of active TB disease. However, it should be noted that IFN-γ variability has important implications for clinical practice and requires caution in interpreting the results to distinguish new infections from nonspecific interindividual variations in cytokine responses.

There are several limitations of our study that must be considered. First, we used the assumption that a positive IGRA is equivalent to LTBI, although it is recognized that there is no gold standard for *M.tb* infection. Second, there was a significant difference between the groups with and without TB contact in terms of age, and the percentage of the youngest study participants (<2 years) was significantly higher in the group of volunteers with TB contact than without TB contact. Given that the development of the immune system is complete when a child reaches the age of 6 or 7, young age may also be a factor in the development of tuberculin hypersensitivity. Another weakness of our study is the lack of repeat IGRA testing in all study participants. IGRAs were assessed in all volunteers at the beginning of the study and were repeated after 8 weeks only if the first result was negative. Given the significant fluctuations in IFN-γ production levels observed in individuals with long-term exposure to *M.tb*, the diagnosis of LTBI should not be based solely on a single IGRA result. However, it should be noted that, despite several limitations and drawbacks of our study, we were able to demonstrate that both TST and IGRA can be used to assess LTBI in TB-exposed children and adolescents undergoing mandatory BCG vaccination in childhood and living in a country with moderate TB exposure. Interpretation of TST results requires caution due to the higher proportion of discordant TST+/IGRA− results, suggesting the influence of reactivity induced by previous BCG vaccination.

## 5. Conclusions

In conclusion, both TST and IGRA can be used as screening tests for BCG-vaccinated children and adolescents exposed to contagious TB. Although IGRAs have a significant logistical advantage over TST, it should be remembered that the results of both tests should be evaluated in light of prevalence and exposure, and their results should never be considered alone. Diagnostic and treatment methods should always be based on a thorough clinical evaluation with attention to risk assessment.

## Figures and Tables

**Table 1:** Demographic characteristics of the study cohort.

Characteristic	Total	TB Contact Children	No TB Contact Children
Total, *n*	235	115	120
Age (years)			
median	7	6 *	8 *
IQR	4–17	2–17	5–17
Age range, *n* (%)			
<2 years	41 (17%)	30 (26%) **	11 (9%) **
3–5 years	44 (19%)	19 (16%)	25 (21%)
6–10 years	72 (31%)	34 (30%)	38 (32%)
>10 years	78 (33%)	32 (28%)	46 (38%)
Sex			
Girls, *n* (%)	120 (51%)	58 (50%)	61 (51%)
Boys, *n* (%)	115 (49%)	57 (50%)	59 (49%)

Abbreviations: IQR—interquartile range; *n*—number; TB—tuberculosis. * *p* = 0.01 (Mann–Whitney U test); ** *p* = 0.0006 (χ^2^ test).

**Table 2:** Comparison of the tuberculin skin test (TST) and interferon-gamma release assay (IGRA) results among TB contact and no TB contact volunteers.

Characteristic	Total	TB Contact Children	No TB Contact Children
Total, *n*	235	115	120
TST, *n* (%)			
negative (<10 mm)	146 (62%)	47 (41%)	99 (82%)
positive (≥10 mm)	89 (38%)	68 (59%) *	21 (18%) *
TST range, *n* (%)			
<5 mm	113 (48%)	29 (25%) **	84 (70%) **
5–9 mm	33 (14%)	18 (16%)	15 (12%)
10–14 mm	62 (26%)	46 (40%) ***	16 (13%) ***
>15 mm	27 (12%)	22 (19%) ****	5 (5%) ****
IGRA, *n* (%)			
negative	161 (68%)	41 (36%) *****	120 (100%) *****
positive	74 (32%)	74 (64%)	0 (0%)

Abbreviations: IGRA—interferon-gamma release assay; *n*—number; TB—tuberculosis; TST—tuberculin skin test. * *p* = 0.00001; ** *p* = 0.00001 *** *p* = 0.00001; **** *p* = 0.0001; ***** *p* = 0.00001. The nonparametric chi-square test or Fisher’s exact test was used to assess the statistical significance of the differences in frequency.

**Table 3:** Sensitivity, specificity, positive predictive value (PPV), and negative predictive value (NPV) for TST in correctly identifying IGRA positivity in TB contact volunteers.

TB Contact Group				
	IGRA-Positive	IGRA-Negative	∑	
TST-positive	62	12	84	PPV 84%
TST-negative	6	35	41	NPV 85%
∑	68	47	115	
	Sensitivity 91%	Specificity 75%		

**Table 4:** Sensitivity, specificity, positive predictive value (PPV), and negative predictive value (NPV) for TST in correctly identifying IGRA positivity in no TB contact volunteers.

No TB Contact Group				
	IGRA-Positive	IGRA-Negative	∑	
TST-positive	0	21	21	PPV 0%
TST-negative	0	99	99	NPV 100%
∑	0	120	120	
	Sensitivity 100%	Specificity 83%		

**Table 5:** Combination of the tuberculin skin test (TST) and interferon-gamma release assay (IGRA) results.

Group	TST Result	IGRA Result *n* (%)		Raw Agreement (%)
		Positive	Negative	
Total (*n* = 235)	Positive	62 (26%)	27 (12%)	83%
	Negative	12 (5%)	134 (57%)	
TB contact children (*n* = 115)	Positive	62 (54%) *	6 (5%)	84%
	Negative	12 (11%)	35 (30%) **	
No TB contact children (*n* = 120)	Positive	0 (0%)	21 (18%)	82%
	Negative	0 (0%)	99 (82%) **	

Abbreviations: IGRA—interferon-gamma release assay; TB—tuberculosis; TST—tuberculin skin test. * *p* < 0.00001; ** *p* < 0.00001. The nonparametric chi-square test or Fisher’s exact test was used to assess the statistical significance of the differences in frequency. Utilizing the quantified agreement with Cohen’s kappa coefficients, the degree of agreement between the TST and IGRA test results was determined.

**Table 6:** Combination of the tuberculin skin test (TST) and interferon-gamma release assay (IGRA) results by age range.

Group/Combination	Age Range *n* (%)			
	<2 Years	Years	6–10 Years	>10 Years
Total (*n* = 235)				
TST+/IGRA+	6 (9%) *	10 (16%)	17 (27%)	29 (48%) *
TST+/IGRA−	6 (22%)	8 (30%)	5 (18%)	8 (30%)
TST−/IGRA+	1 (8%)	5 (42%)	5 (42%)	1 (8%)
TST−/IGRA−	28 (21%)	21 (16%)	45 (33%)	40 (30%)
TB contact children (*n* = 115)				
TST+/IGRA+	6 (10%)	10 (16%)	17 (27%)	29 (47%)
TST+/IGRA−	5 (83%)	0 (0%)	1 (17%)	0 (0%)
TST−/IGRA+	1 (8%)	5 (42%)	5 (42%)	1 (8%)
TST−/IGRA−	18 (51%)	4 (12%)	11 (31%)	2 (6%)
no TB contact children (*n* = 120)				
TST+/IGRA+	0 (0%)	0 (0%)	0 (0%)	0 (0%)
TST+/IGRA−	1 (5%)	8 (38%)	4 (19%)	8 (38%)
TST−/IGRA+	0 (0%)	0 (0%)	0 (0%)	0 (0%)
TST−/IGRA−	10 (10%)	17 (17%)	34 (34%)	38 (39%)

Abbreviations: IGRA—interferon-gamma release assay; TB—tuberculosis; TST—tuberculin skin test. * *p* = 0.01. The nonparametric chi-square test or Fisher’s exact test was used to assess the statistical significance of the differences in frequency.

**Table 7:** The concentration of IFN-γ produced in response to specific *M.tb* antigens in the IGRA cultures in the groups of TB and LTBI volunteers.

IFN-γ (IU/mL)							
TB				LTBI			
NIL	TB1	TB2	Mitogen	NIL	TB1	TB2	Mitogen
0.23 ± 0.19	1.48 ± 2.44 *	1.84 ± 2.19 **	7.76 ± 3.53	0.18 ± 0.08	3.51 ± 2.80 *	4.38 ± 3.16 **	8.42 ± 2.52

Abbreviations: NIL—negative control; IFN-γ—interferon-gamma; IU—international unit; TB1—TB1 Antigen Tube, TB2—TB2 Antigen Tube; * *p* = 0.0005, ** *p* = 0.0007. The nonparametric Mann–Whitney U test was used to assess the statistical significance of differences in IFN-γ concentrations in TB1 and TB2 blood cultures between TB and LTBI groups.

## Data Availability

Not applicable.

## Supplementary Materials

The following supporting information can be downloaded at https://www.mdpi.com/article/10.3390/vaccines11020387/s1: Table S1. Characteristics of children with active TB disease; Figure S1. The mean level of IFN-γ produced by leukocytes following stimulation with *M.tb* antigens in TB1 (white bars) and TB2 (gray bars) QuantiFERON TB Plus whole-blood cultures in the groups of TB contact and no TB contact volunteers with negative (TST−) or positive (TST+) reaction to tuberculin; Figure S2. Mean levels of IFN-γ produced by leukocytes after stimulation with *M.tb* antigens in TB1 (white bars) and TB2 (gray bars) QuantiFERON TB Plus whole-blood cultures in the groups of volunteers with negative (TST−) or positive (TST+) reaction to tuberculin by age group; Figure S3. Correlation between IFN-γ levels produced by leukocytes after stimulation with *M.tb* antigens in TB1 (A) and TB2 (B) QuantiFERON TB Plus whole-blood cultures and TST induration size.
