# Supplementary material for: The Interferon-Gamma Release Assay versus the Tuberculin Skin Test in the Diagnosis of Mycobacterium tuberculosis Infection in BCG-Vaccinated Children and Adolescents Exposed or Not Exposed to Contagious TB
Source: Vaccines (Basel). 2023 Feb 7;11(2):387. doi: 10.3390/vaccines11020387 (PMC9961142; doi:10.3390/vaccines11020387)
Supplement: Supplementary file 1 [file vaccines-11-00387-s001.zip › vaccines-2163635-supplementary.pdf]

vaccines

MDPI

Supplementary Table S1. Characteristics of children with active TB disease

Age (years)	Sex	TST		IGRA					Chest radiograph	Material *M.tb* confirmation
		result	induration (mm)	result	IFN-γ (IU/ml)					
					Nil	TB1	TB2	Mitogen		
1	F	+	25	+	0.9	>10	>10	>10	abnormal, no cavities	gastric aspirate, *M.tb*-culture+
16	F	+	20	+	0.35	3.93	>10	>10	abnormal, no cavities	bronchoaspirate, *M.tb*-culture+
16	F	+	18	+	0.16	0.79	0.93	>10	abnormal, no cavities	gastric aspirate, *M.tb*-culture+
16	M	+	13	+	0.17	0.49	0.60	9.98	abnormal, no cavities	bronchoaspirate, *M.tb*-culture+
6	F	-	4	-	0.12	0.10	0.08	>10	abnormal, no cavities	gastric aspirate, *M.tb*-culture+
15	F	+	10	+	0.13	1.04	0.84	>10	abnormal, cavitary disease	gastric aspirate, *M.tb*-culture+
8	F	+	18	+	0.22	1.24	1.29	8.68	abnormal, no cavities	gastric aspirate, *M.tb*-culture+
16	F	+	16	+	0.21	0.61	0.70	1.13	abnormal, cavitary disease	gastric aspirate, *M.tb*-culture+
11	M	+	15	+	0.51	>10	>10	>10	abnormal, no cavities	gastric aspirate, *M.tb*-culture+
16	F	+	18	+	0.29	0.81	0.88	>10	abnormal, no cavities	gastric aspirate, *M.tb*-culture+
16	M	+	20	+	0.21	0.48	0.56	1.85	abnormal, no cavities	gastric aspirate, *M.tb*-culture+
15	F	+	20	+	0.14	1.82	1.03	>10	abnormal, no cavities	gastric aspirate, *M.tb*-culture+
15	F	-	0	-	0.19	0.24	0.21	>10	abnormal, no cavities	gastric aspirate, *M.tb*-culture+
14	M	+	10	+	0.20	0.51	0.59	>10	abnormal, no cavities	gastric aspirate, *M.tb*-culture+
17	M	-	0	-	0.28	0.30	0.35	5.37	abnormal, no cavities	gastric aspirate, *M.tb*-culture+
17	M	+	17	+	0.08	0.42	0.51	0.67	abnormal, no cavities	bronchoaspirate, *M.tb*-culture+

Abbreviations: F – female; IGRA – interferon-gamma release assay; IU – international units; M – male; *M.tb* – *Mycobacterium tuberculosis*; TST – tuberculin skin test.

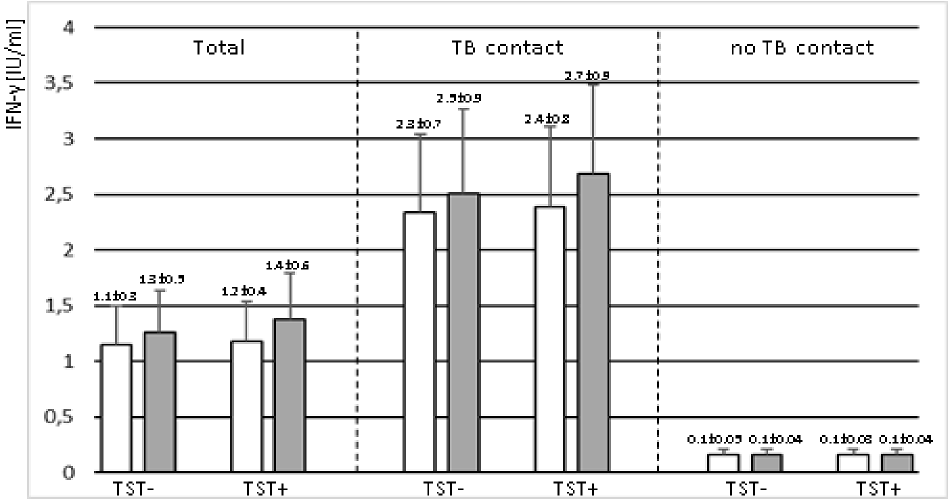

Supplementary Figure S1. The mean level of IFN-γ produced by leukocytes following stimulation with *M.tb* antigens in TB1 (white bars) and TB2 (grey bars) QuantiFERON TB Plus whole blood cultures in the groups of TB contact and no TB contact volunteers with negative (TST-) or positive (TST+) reaction to tuberculin.

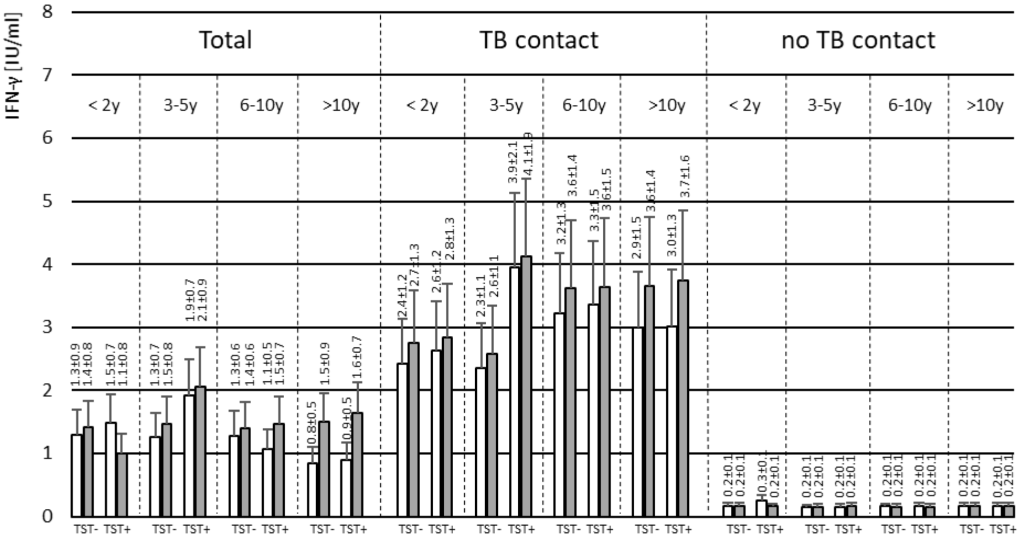

Supplementary Figure S2. Mean levels of IFN-γ produced by leukocytes after stimulation with *M.tb* antigens in TB1 (white bars) and TB2 (grey bars) QuantiFERON TB Plus whole blood cultures in the groups of volunteers with negative (TST-) or positive (TST+) reaction to tuberculin by age group.

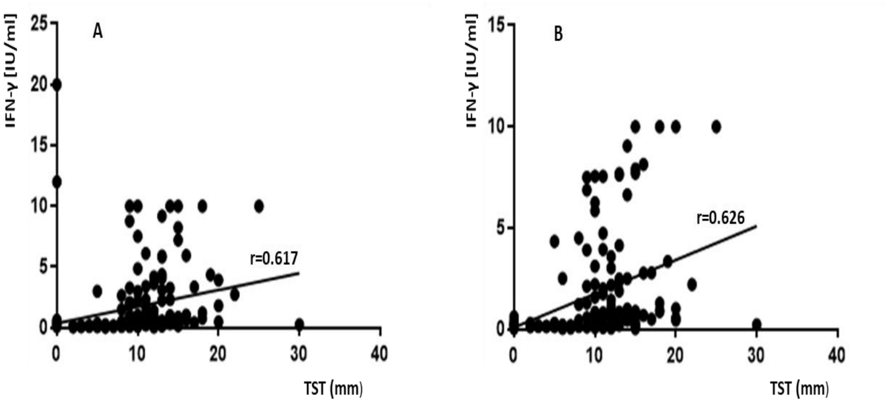

Supplementary Figure S3. Correlation between IFN-γ levels produced by leukocytes after stimulation with *M.tb* antigens in TB1 (A) and TB2 (B) QuantiFERON TB Plus whole blood cultures and TST induration size.
